# Supplementary material for: Methanogenesis at High Temperature, High Ionic Strength and Low pH in the Volcanic Area of Dallol, Ethiopia
Source: Microorganisms. 2021 Jun 6;9(6):1231. doi: 10.3390/microorganisms9061231 (PMC8228321; doi:10.3390/microorganisms9061231)
Supplement: Supplementary file 1 [file microorganisms-09-01231-s001.zip › Table S1.pdf]

Table S1. Carbon ($\delta^{13}C_{CH4}$) stable isotopic composition of the methane analyzed in the target samples.

	**Sample**	**$\delta^{13}C_{CH4}$ (‰)***
Yellow Fumaroles	$D6_a$	-32.8 ± 0.3
	$D6_b$	-32.6 ± 0.7
	D6S	-37.0 ± 0.6
Blackish White/Grayish Fumaroles	$FNA1_a$	-33.1 ± 0.6
	$FNA1_b$	-33.1 ± 0.7
	FNA2	-35.8 ± 0.6

*Values are means ± standard deviations
