# Supplementary figures and images for: Methanogenesis at High Temperature, High Ionic Strength and Low pH in the Volcanic Area of Dallol, Ethiopia
Source: Microorganisms. 2021 Jun 6;9(6):1231. doi: 10.3390/microorganisms9061231 (PMC8228321; doi:10.3390/microorganisms9061231)

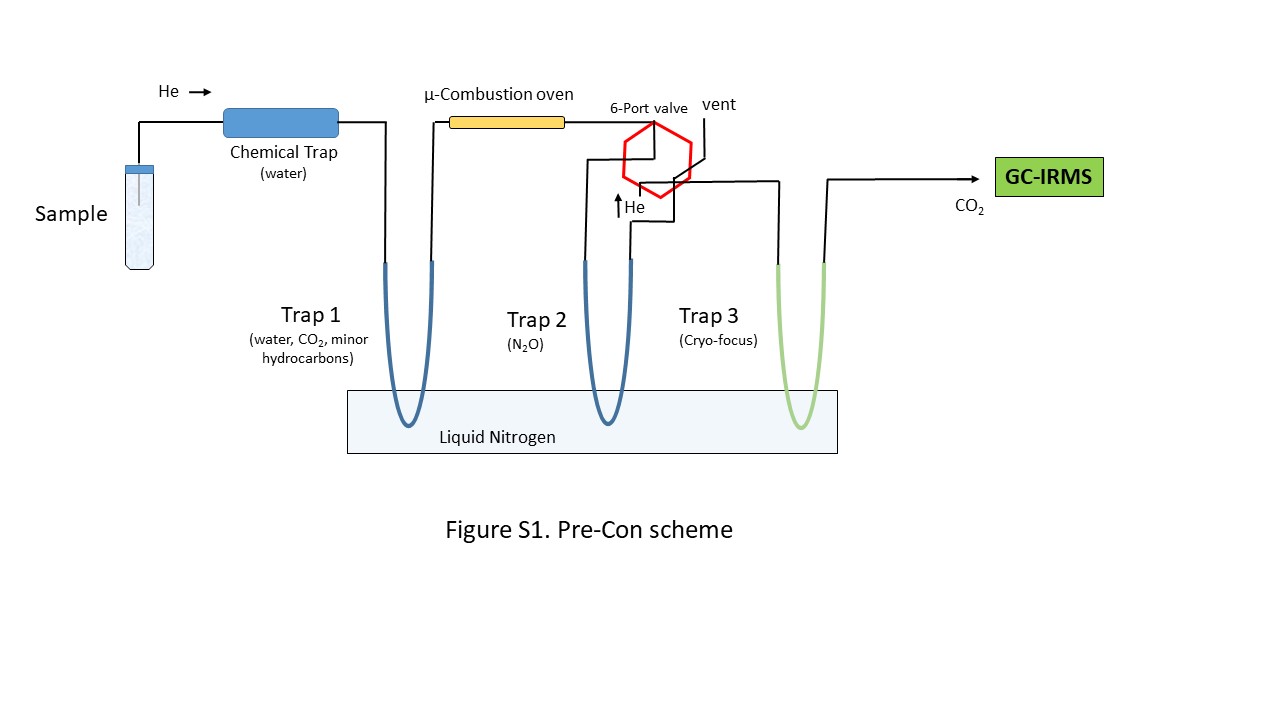

Supplement: Supplementary file 1 [file microorganisms-09-01231-s001.zip › Figure S1.jpg]
